# Understanding the effect of occupational stress on sleep quality in firefighters: the modulating role of depression and burnout

**DOI:** 10.1007/s00420-024-02104-9

**Published:** 2024-10-22

**Authors:** Amir Hossein Khoshakhlagh, Saleh Al Sulaie, Rosanna Cousins, Saeid Yazdanirad, Fereydoon Laal

**Affiliations:** 1 Department of Occupational Health Engineering, School of Health, Kashan University of Medical Sciences, Kashan, Iran; 2 Department of Industrial Engineering, College of Engineering in Al-Qunfudah, Umm Al-Qura University, Makkah, 21955 Saudi Arabia; 3 Department of Psychology, Liverpool Hope University, Hope Park, Liverpool, L16 9JD UK; 4 School of Health, Shahrekord University of Medical Sciences, Shahrekord, Iran; 5 Department of Occupational Health Engineering, Birjand University of Medical Sciences, Birjand, Iran

**Keywords:** Fire and rescue services, Work-related stress, Post-traumatic stress, SIT benchmarks, Sleep problems, Structural equation modelling

## Abstract

**Objectives:**

Sleep quality of firefighters can be negatively affected by occupational stressors. A cross-sectional investigation was conducted to understand how work-related stress, post-traumatic stress, burnout and depression collectively contribute to sleep quality.

**Methods:**

Professional firefighters in Northern Iran completed a survey comprised of demographic information, the Pittsburgh Sleep Quality Index, HSE’s Stress Indicator Tool, the Posttraumatic Stress Disorder Checklist, Maslach’s Burnout Inventory, and the Beck Depression Inventory during a work rest break. Data were analysed using structural equation modelling.

**Results:**

Mean age of the 2339 firefighters who completed the survey was 32.30 (5.74) years. Most experienced poor sleep quality, scoring above the established cut-off of 5. Levels of work-related stress, post-traumatic stress, burnout and depression were high. Fit indices of the final theoretical model were all adequate: the obtained and adjusted goodness-of-fit indices were 0.925 and 0.917 respectively. Comparative, and incremental fit indices were 0.946 and 0.948 respectively. Root mean squared error of approximation was 0.061. Post-traumatic stress was directly and indirectly related to sleep quality through eight paths, modulated by burnout variables and depression. Work-related stress was negatively related to sleep quality through four paths modulated, by burnout variables and depression.

**Conclusions:**

The findings illustrate the complex relationships of work-related stress and post-traumatic stress and sleep quality. High levels of poor sleep quality in this occupation emphasise the need to develop targeted and sustainable interventions to manage occupational stressors, burnout and depression to improve sleep quality in firefighters.

## Introduction

Poor sleep quality and consequent tiredness and fatigue has a negative impact on health though a reduction in immune system efficiency, compromised hypothalamus, pituitary and adrenal axis function, and increased blood pressure – all of which can also reduce work performance and disrupt healthy social relationships (Basit et al. 2018; Haack and Mullington 2005). Workers who suffer from poor sleep quality also have an increased risk of accidents, take more medical drugs, and have higher rates of sickness absence, all of which impose costs on employers and on society (Haack and Mullington 2005). One job known to have a high prevalence of sleep disorders and poor sleep quality is firefighting. This occupation is marked by shift working, long working hours, long periods of work with no breaks for nutrition or rest, and prolonged tension to perform operations optimally to ensure no lives are lost (Khoshakhlagh et al. 2023). Firefighting is also an occupation with many inherent work-related stressors (Igboanugo et al. 2021; Payne and Kinman 2019) which can also impact on sleep quality (Iwasaki et al. 2018; Matti et al. 2024), in addition to disruption to circadian rhythms from work rotas (Billings and Focht 2016).

There have been several studies which have confirmed that properties of the firefighters’ job affect their sleep quality and performance. These include a survey of 427 firefighters in Iran which found that almost 70% of participants had poor sleep quality according to the Pittsburgh Sleep Quality Index (PSQI) (Mehrdad et al. 2013). A high prevalence of poor sleep quality in firefighters according to the PSQI has also been found in the US (59%) (Carey et al. 2011), in South Korea (49%) (Lim et al. 2014), and in a subsequent study in Iran (59%) (Abbasi et al. 2018). In the South Korean study, the Odds Ratio (OR) of shift working impacting on sleep quality was 1.58; but there were higher OR for musculoskeletal symptoms (OR = 2.89), and depression (OR = 7.04) (Lim et al. 2014). There was also a significant relationship of sleep quality and depression in the US study (Carey et al. 2011), however for 89% of the sample (*N* = 112) their score on the Beck Depression Inventory did not reach the level that would indicate mild-to-moderate depression. Both depression and shift working were significantly more likely in firefighters with poor sleep quality in the Abbasi et al. (2018) study (*N* = 118), as well as ascertaining that there were relationships of sleep quality and work-related stress, musculoskeletal disorders, and BMI in bivariate correlation analyses. Abbasi et al. performed a linear regression analysis to tease out the independent factors affecting sleep quality which confirmed that musculoskeletal disorders, shift work, and BMI were independent predictors of sleep quality. Work-related stress, as measured by the HSE Stress Indicator Tool (SIT; Cousins et al. 2004), came out of the explanatory model, but, as *P* = 0.06, their null hypothesis that work-related stress had no impact on sleep quality could not be rejected. The authors acknowledged that the analysis was likely to be underpowered, and thus the findings should be treated with caution. Altogether this pointed to a need for further studies to determine the causal factors of poor sleep quality in firefighters.

Besides work-related stress, the work of firefighters provides potentials for post-traumatic stress disorder (PTSD) because of their exposure to traumatic events, which in turn relates to a higher probability of poor sleep quality (Khumtong and Taneepanichskul 2019). There is also evidence that sleep quality mediates suicide risk in firefighters who experience post-traumatic stress symptoms (Healy and Vujanovic 2021). An investigative review of risk factors for PTSD in firefighters found pre-existing sleep disorders, depression, anxiety, work-related stress, and physical symptoms were all risk factors for PTSD (Ryu et al. 2017), all of which indicates a vicious circle of cause and effect is involved with poor sleep quality, depression, work-related stress and post-traumatic stress and negative psychological and physical outcomes. Wagner et al. (2020) reported that most firefighters are exposed to traumatic events on a regular basis, but that substantial differences in study quality, sample type and size, pose problems for obtaining reliable estimates of the prevalence of PTSD in firefighters. In common with Ryu et al. (2017), Wagner et al. (2020) found a relationship of depression and PTSD, and they went on to consider that symptoms of PTSD and depression occur together following exposure to a traumatic event. This hypothesis needs further investigation, including considering whether depression has a modulating role in the manifestation of post-traumatic symptoms.

Previous attempts to understand the role of sleep quality in the manifestation of work-related stress and post-traumatic stress in firefighters have commonly included burnout variables, as there is evidence that burnout is associated with chronic work-related stress among firefighters (Smith et al. 2018), and burnout has been implicated in sleep problems in nurses (Membrive-Jiménez et al. 2022). Burnout has been described as a syndrome of emotional exhaustion, feelings of low personal accomplishment, and depersonalisation in employees working in human services (Maslach and Jackson 1981), and the outcome of many unsuccessful attempts to cope with stressful conditions (Khoshakhlagh et al. 2023; Ryu et al. 2017). Hence this variable should also be included in an examination of predictors of sleep problems in firefighters. Previous recognition of the importance of burnout led to the inclusion of the Maslach Burnout Inventory (Maslach and Jackson 1981) in a survey to examine sleep disorder risk and mental health outcomes in North American firefighters (Wolkow et al. 2019). Sleep quality was measured using five different self-assessments and mental health measures included self-reports of depression, anxiety, PTSD and burnout. Outcomes from the 6307 firefighters indicated that 38% screened positive for any sleep disorder, and 48% reported high burnout, 6% had a current diagnosis of depression, and 1.8% had PTSD. Interestingly, while the amount of co-morbidity of sleep disorders, mental health conditions and burnout was small, poor sleep and mental health were associated with high levels of burnout, and that sleep during overnight work mediated the risk of sleep disorders and burnout.

Altogether, the extant literature confirmed that poor sleep quality is common in firefighters, but the evidence on the contribution of burnout, work-related stress, PTSD, and depression variables to understanding sleep quality in firefighters was mixed. Therefore, the aim of this study was to examine the dynamic relationships of work-related stress, post-traumatic stress, burnout and depression on sleep quality in firefighters using structural equation modelling. Objectives were to understand the contribution of work-related stress and post-traumatic stress to sleep quality, and whether these variables could be modulated by burnout and by depression. Altogether, this would progress the literature in this area, and support development of interventions to prevent and treat the high level of sleep problems in firefighters.

## Methods

### Design and participants

**Fig. 1:**
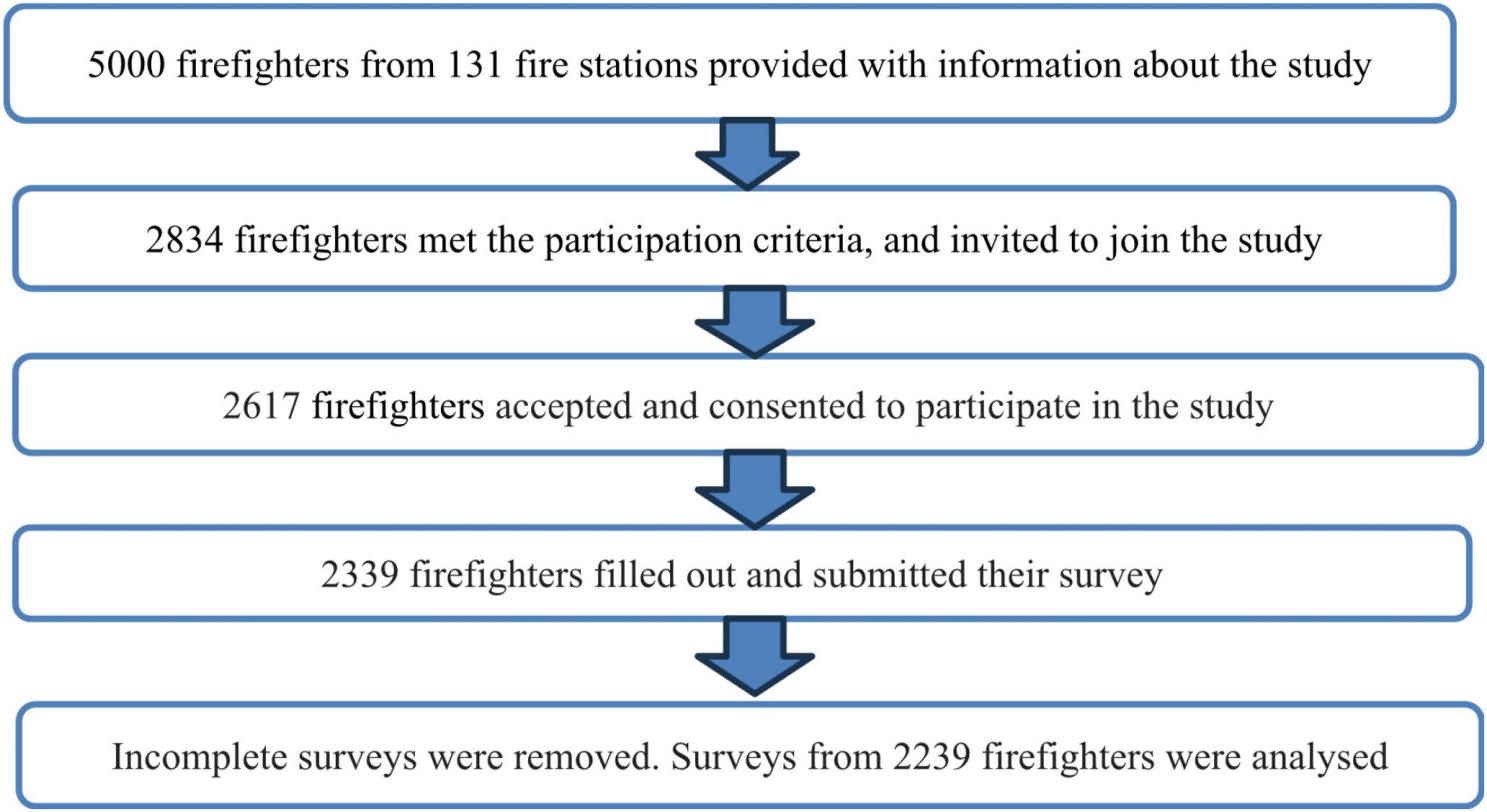
Flow diagram of recruitment to the study

This cross-sectional survey study was conducted in Northern Iran in 2022. Ethical approval was granted by the Research Ethics Committee of Birjand University of Medical Sciences (No. IR.BUMS.REC.1401.195). The study was completed in accordance with the principles of the Declaration of Helsinki. The study was advertised in 131 fire stations in Northern Iran. To collect a sufficiently large sample, and eliminate potentials for bias in sampling, all 5000 professional firefighters in Northern Iran were given information about the study with an invitation to join the study if they met the participation criteria. The inclusion criterion was at least one year of work experience; exclusion criteria were unwillingness to complete the survey and a history of mental or physical disease. As shown in Fig. 1, just over half the firefighters (56.58%) were eligible and provided informed consent. 2617 firefighters were provided with a self-report hard copy of the survey to complete in work time during a rest break. After removing incomplete surveys, a final sample of 2339 firefighters included in the analyses. This response rate was 82.53% of those invited to be participants in the study.

### Materials

The survey comprised a section to collect demographical information, and five standard questionnaires, as described below. The demographic information section included questions regarding age, education level, body mass index – which were categorized, and three dichotomous questions for marital status (married / single), second job (yes / no) and smoking status (yes / no).

#### Pittsburgh sleep quality index

The Pittsburgh Sleep Quality Index (PSQI; Buysse et al. 1989) is a 19-item self-report questionnaire to investigate the various aspects of sleep quality in the past month. Items are scored on a 4-point Likert scale from 0 (not during the past month) to 3 (three or more times a week) then grouped to provide seven component scores (also scored 0 to 3) and a Global PSQI score (range 0 to 21). Higher scores indicate poorer sleep quality. According to Buysse et al. (1989), a Global PSQI score > 5 can identify poor sleepers with a sensitivity of 89.6% and a specificity of 86.5%. The seven components are: subjective sleep quality, sleep latency, sleep duration, habitual sleep efficiency, sleep disturbances, use of sleep medication, and daytime dysfunction. The Persian version (PSQI-P) used in the study (Moghaddam et al. 2012) has acceptable reliability (Cronbach’s alpha = 0.77).

#### Work-related stress

The HSE Stress Indicator Tool (SIT; Cousins et al. 2004) was used to assess work-related stress. This is a 35-item questionnaire with seven subscales: Demands (8 items), Control (6 items), Managerial support (5 items), Peer support (4 items), Relationships (4 items), Role (5 items), and Change (3 items). Each item is answered using a 5-point forced-choice agreement or frequency Likert Scale (scored 1–5). Higher SIT scores indicated a lower risk of work-related stress. Cousins et al. reported high internal consistency for each of the seven subscales, with Cronbach’s alpha coefficients ranging from 0.78 (Relationships) to 0.89 (Demands). The Persian language version used in this study is valid and reliable (Azad-Marzabadi et al. 2011; Mirzaei et al. 2022). Besides collecting raw scores, the data was also standardised (mean scores divided by number of items on the scale) to allow comparison with the latest SIT industry specific benchmarking data for emergency services (HSE 2023). Mean scores lower than the benchmark are indicative of work-related stress in employees in that stressor.

#### Posttraumatic stress disorder checklist

To assess PTSD symptoms, the Posttraumatic Stress Disorder Checklist – Civilian (PCL-C) was used. This is a 17-item questionnaire based on DSM-IV B, C, & D criteria for PTSD symptoms designed for use in non-military samples (Weathers and Ford 1996). The PCL-C asks about symptoms in relation to non-specific stressful experiences which simplifies assessment in populations that may have experienced multiple traumas. Hence this checklist has previously been used for assessing post-traumatic symptoms in professional firefighters (e.g. Del Ben et al. 2006; Kim et al. 2018; Marcelino and Gonçalves 2012). Items address the three key symptoms of PTSD: Re-experiencing (5 items), Numbing/avoidance (7 items), and Hyperarousal (5 items). Participants were asked to rate their distress regarding the item symptom in the past month using a 5-point Likert scale (1 = not at all to 5 = extremely). Total post-traumatic stress symptom severity is calculated by adding the three subscale scores, providing a range of 17–85. There is no absolute method for determining cut-offs for the considering possible PTSD, and perusal of the literature indicates that there are a variety of cut-offs that have been utilized for the PLC-C according to population (Gelaye et al. 2017; Vera-Villarroel et al. 2011). Nevertheless, it is considered that the diagnostic efficiency is good (Blanchard et al. 1996; Weathers and Ford 1996). The Persian version used in this study has been confirmed as reliable and valid (Zarei et al. 2016).

#### Maslach burnout inventory

The Maslach Burnout Inventory (MBI; Maslach and Jackson 1981) has been validated in many studies and translated into several languages including Persian (Motaghedi et al. 2016). The MBI has 22 items, and three scales: emotional exhaustion (9 items), depersonalization (5 items), and personal accomplishment (8 items). Each item is measured on a 7-point Likert scale from 0 (never) to 6 (every day), a scale score derived from the sum of the item scores. Higher scores indicate higher levels of burnout.

#### Beck depression inventory II

The BDI-II-Persian was used (Ghassemzadeh et al. 2005). This is a reliable and valid translation of the original self-report 21-item Beck Depression Inventory. Items examine the severity of symptoms in the previous two weeks using a 4-point scale from 0 (absent or mild) to 3 (severe). Item scores are summed to provide a total score (range 0 to 63).

### Data analysis

Descriptive statistics were analysed using IBM SPSS Statistics (version 26). Normality was examined by skewness and kurtosis curves. The statistical distribution of all parameters was normal therefore the correlation coefficients were computed using Pearson’s tests. Structural Equation Modelling was used to develop a theoretical model using Analysis of Moment Structures (AMOS) version 26 (USA, IBM Inc). Structural Equation Modelling is a powerful multivariate analysis technique that can measure relationships between observed and latent variables, and test complex theoretical models with one analysis. Nevertheless, evaluation of the fit of a theoretical model to the collected data is not simple as it is necessary to consider multiple criteria simultaneously. To determine the value of the structural equation models a large variety of indices have been developed. In line with normal practice, we used several conventional indices in the estimation procedure and evaluation of the final fit of the model. These included observed and adjusted goodness-of-fit indices, the comparative fit index, the normed fit index, the incremental fit index, all of which have no absolute value for model acceptance. A value of 1 indicates a perfect fit, but there is a ‘rule of thumb’, if not universally agreed, that models are acceptable if a fit index if higher than 0.90 (Schermelleh-Engel et al. 2003). We also measured root mean square error of approximation: a value ≤ 0.05 is a good fit, and values between 0.05 and 0.08 are adequate (Browne and Cudeck 1993), and the normed chi-square (*X*^*2*^*/df*) although this is a statistic greatly affected by sample size and number of observations. As our sample size was large, this was included. *X*^*2*^*/df* ≤ 2 is considered acceptable. To examine the potential sequential modulating effects of depression and burnout in the relationship of the two types of firefighter stress (PTSD and job stress) and sleep quality, bootstrapping was used to assess indirect effects. Following Cohen (1988), effect coefficients < 0.10 present a small effect, values around 0.30 indicate a medium effect, and values of 0.50 or more, a large effect.

## Results

The mean (SD) values of age and body mass index of the 2239 firefighters who completed the survey were 32.30 (5.74) years and 26.64 (6.39) kg/m^2^, respectively. All participants were male. This is normal in many countries, including Iran. Regarding age: most participants were under 40 years (38.8% were under 30 years, 55.1% were 30–40 years, 5.1% were 41–50, and 0.8% were over 50 years). Most firefighters were educated to at least diploma level (75.2%), did not have a second job (55.8%), were married (66.9%), and did not smoke (77.9%). There was no difference in sleep quality according to the demographic variables.

Results of the studied variables in terms of range, means and SIT Emergency Services benchmarks are presented in Table 1. Sleep quality scores, as indicated by the PSQI-P ranged from 2 to 21 and the mean value of 9.72 (5.09) which confirmed that most of the sample experienced poor sleep, as identified by the established cut-off of > 5 presented by Buysse et al. (1989). Regarding work-related stress, standardised means were below HSE’s Emergency Services benchmarks for all stressor areas.

**Table 1:** Descriptive information of the studied variables (*N* = 2239)

Variable		Range of scores	Raw mean (SD)	Standardised mean	Emergency services mean / benchmark
Work-related stress (SIT)	Demands	9–37	24.60 (5.77)	3.08	3.24 / 3.58
	Control	6–30	18.23 (4.74)	3.04	3.27 / 3.45
	Managerial support	5–25	15.55 (4.12)	3.11	3.74 / 3.92
	Peer support	4–20	12.77 (3.54)	3.19	3.93 / 4.04
	Relationships	4–20	12.90 (3.88)	3.22	4.02 / 4.16
	Role	5–25	16.05 (5.63)	3.21	4.13 / 4.26
	Change	3–15	9.41 (2.75)	3.14	3.06 / 3.30
Post-traumatic stress (PCL-C)		17–85	41.54 (19.67)		
Burnout (MBI)	Emotional exhaustion	0–53	22.29 (11.82)		
	Depersonalization	2–27	11.34 (6.37)		
	Personal accomplishment	3–48	26.23 (8.18)		
Depression (BDI-II-Persian)		0–51	16.16 (12.85)		
Sleep quality (PSQI-P)	Subjective sleep quality	0–3	1.03 (1.11)		
	Sleep latency	0–3	1.33 (0.84)		
	Sleep duration	0–3	1.28 (1.18)		
	Habitual sleep efficiency	0–3	1.35 (1.18)		
	Sleep disturbances	0–3	1.73 (0.81)		
	Use sleep medication	0–3	1.45 (0.96)		
	Daytime dysfunction	0–3	1.56 (0.96)		
	Total	2–21	9.72 (5.09)		

As shown in Table 2, there were significant correlations among all variables and sleep quality scores (*P* < 0.01). It was therefore appropriate to include all the variables in the structural equation model to fully explore their predictive value and modulating effects for understanding sleep quality in firefighters. Of the work-related stress dimensions, (high) demands had the highest relationship with (poor) sleep quality (*r* = -0.65). Among the burnout dimensions, depersonalization had the strongest correlation with (poor) sleep quality (*r* = 0.70). Among all the measured variables, the highest correlation coefficient was between post-traumatic stress and sleep quality (*r* = 0.80) which is indicative of a very strong link between PTSD and poor sleep.

**Fig. 2:**
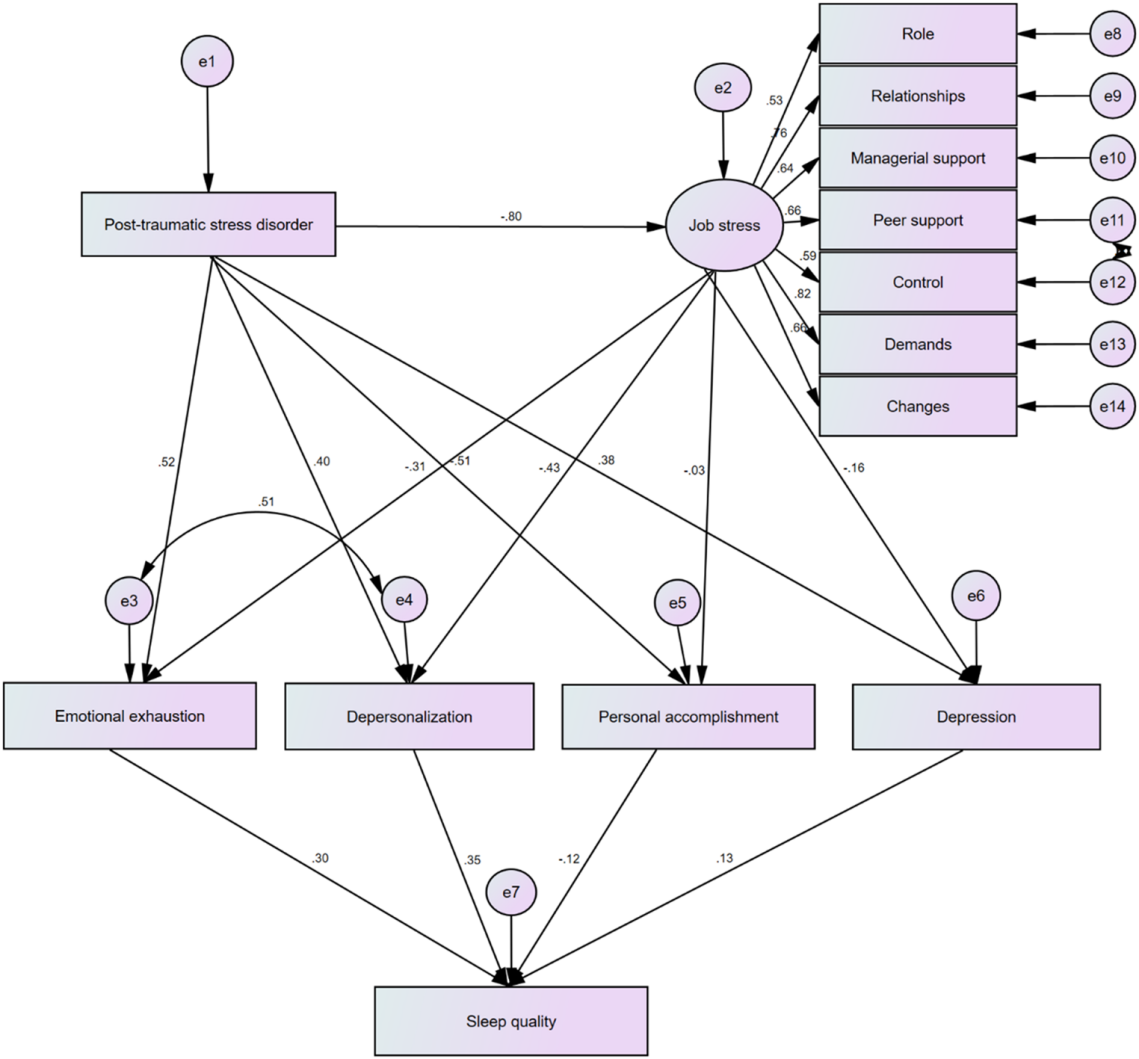
Model of the relationships among the studied variables with path coefficients

Figure 2 shows the results of the structural equation modelling: the final theoretical model of the relationships between the studied variables. The purpose of the model is to provide a statistical statement about the relationship, variation and covariation of the measured variables, resolving the common problem of collinearity that is found with related variables. It is the best representation of the observed data to interpret cause of sleep quality in firefighters. The fit indices were all sufficient to confirm trustworthiness of the model. Regarding absolute fit indices: The obtained goodness-of-fit index was 0.925 and adjusted goodness-of-fit index 0.917 – both greater that the minimum 0.90. The comparative fit index was 0.946, the incremental fit index was 0.948, and the normed fit index was 0.906 – again all > 0.90. The root mean squared error of approximation was 0.061 – indicating an adequate fit. The normed chi-square was 1.98 which also provides confidence in the final model.

**Table 2:** Correlation matrix of the studied variables (*N* = 2239)

	Variable	1	2	3	4	5	6	7	8	9	10	11	12
1	Role	-											
2	Relationships	0.24^**^	-										
3	Managerial support	0.41^**^	0.41^**^	-									
4	Peer support	0.67^**^	0.41^**^	0.53^**^	-								
5	Control	0.68^**^	0.31^**^	0.49^**^	0.69^**^	-							
6	Demands	0.37^**^	0.69^**^	0.50^**^	0.46^**^	0.41^**^	-						
7	Change	0.35^**^	0.49^**^	0.57^**^	0.50^**^	0.48^**^	0.52^**^	-					
8	Post-traumatic stress	-0.39^**^	-0.69^**^	-0.45^**^	-0.51^**^	-0.43^**^	-0.68^**^	-0.46^**^	-				
9	Emotional exhaustion	-0.34^**^	-0.63^**^	-0.43^**^	-0.43^**^	-0.39^**^	-0.62^**^	-0.44^**^	0.77^**^	-			
10	Depersonalization	-0.39^**^	-0.63^**^	-0.43^**^	-0.46^**^	-0.40^**^	-0.63^**^	-0.45^**^	0.74^**^	0.81^**^	-		
11	Personal accomplishment	< 0.01	0.34^**^	0.26^**^	0.14^**^	0.10^**^	0.30^**^	0.29^**^	-0.48^**^	-0.46^**^	-0.45^**^	-	
12	Depression	-0.26^**^	-0.42^**^	-0.25^**^	-0.29^**^	-0.29^**^	-0.36^**^	-0.29^**^	0.51^**^	0.42^**^	0.44^**^	-0.24^**^	-
13	Sleep quality	-0.44^**^	-0.64^**^	-0.50^**^	-0.51^**^	-0.46^**^	-0.65^**^	-0.47^**^	0.80^**^	0.69^**^	0.70^**^	-0.44^**^	0.43^**^

** *P <* 0.01; *P < 0.05

Table 3 reports the direct and modulating effect coefficients of depression and burnout on sleep quality in terms of the primary independent variables post-traumatic stress and job stress. Post-traumatic stress affected sleep quality through eight paths, with small modulating effects of depression and small to medium modulating effects through burnout. Work-related stress impacted sleep quality through four paths. The modulating effects of burnout and depression were generally small. Among burnout dimensions, depersonalization had highest direct effect on sleep quality.

**Table 3:** Direct and modulating effects of depression and burnout (in the relationships of post-traumatic stress and work-related stress) on sleep quality (*N* = 2239)

Variable	Effect *
Post-traumatic stress → work-related stress → emotional exhaustion → sleep quality	0.08
Post-traumatic stress → work-related stress → depersonalization → sleep quality	0.12
Post-traumatic stress → work-related stress → personal accomplishment → sleep quality	0.003
Post-traumatic stress → work-related stress → depression → sleep quality	0.02
Post-traumatic stress → emotional exhaustion → sleep quality	0.16
Post-traumatic stress → depersonalization → sleep quality	0.14
Post-traumatic stress → personal accomplishment → sleep quality	0.06
Post-traumatic stress → depression → sleep quality	0.05
Work-related stress → emotional exhaustion → sleep quality	− 0.10
Work-related stress → depersonalization → sleep quality	− 0.15
Work-related stress → personal accomplishment → sleep quality	− 0.004
Work-related stress → depression → sleep quality	− 0.02
Emotional exhaustion → sleep quality	0.30
Depersonalization → sleep quality	0.35
Personal accomplishment → sleep quality	− 0.12
Depression → sleep quality	0.13

* Following Cohen (1988), effect coefficients < 0.10 present a small effect, values around 0.30 indicate a medium effect, and values of 0.50 or more, a large effect

## Discussion

The aim of this research was to investigate how work-related stress, post-traumatic stress, burnout and depression collectively contribute to sleep quality in firefighters. Sleep quality was confirmed as poor in most of the sample. The mean PSQI-P score was well above the established cut-off score of Buysse et al. (1989). Structural equation modelling confirmed the significance of work-related stress and post-traumatic stress in poor sleep quality and that this was modulated through burnout and depression in four and eight paths respectively. Whilst the extant literature had pointed to high levels of poor sleep quality in this occupational group, accounting for the significant contribution of work-related stress, post-traumatic stress, burnout and depression was less clear. This research has added to the literature in illustrating direct and indirect relationships involved in understanding sleep quality.

The extant literature has identified the importance of job stress, PTSD, burnout and depression in understanding sleep quality in firefighters, however studies used small sample sizes and thus had insufficient power to provide robust findings. This study used a very large sample of firefighters giving confidence to the findings. Additionally, generally the methodology of previous studies lacked the sophisticated analyses required to understand the patterns of correlation and covariance among variables of interest and sleep quality. In this study we illustrated how primary predictors (job stress and post-traumatic stress) may be modulated by consequential variables (burnout and depression) in their impact on sleep quality. That is, besides their direct relationships with sleep quality, burnout and depression were found to be involved in sleep quality through significantly modulating the influence of work-related stress and post-traumatic stress on sleep quality.

The survey data clearly showed that levels of work-related stress were high in the firefighters, even when aligned to SIT benchmarks for all emergency services (HSE 2023). Looking at the component parts of job stress, we confirmed that all seven stressors in the robust Stress Indicator Tool (Cousins et al. 2004) were negatively related to sleep quality, confirming assertions of this relationship in previous studies (Abbasi et al. 2018; Khoshakhlagh et al. 2023; Payne and Kinman 2019). There were also strong bilateral relationships of post-traumatic stress, the three burnout measures, depression and sleep quality. Good management of work-related stress beyond compliance with legal standards is beneficial in all occupations (Cousins et al. 2004). Work-related stress is an important factor in poor health, including poor sleep quality, which in turn begets reduced workability and more illness (Kalimo et al. 2000; Khoshakhlagh et al. 2023). In this study, the obtained theoretical model implicates work-related stress in sleep quality among firefighters, and that this can be modulated by burnout and depression. As such, regular risk assessments should seek to identify work-related stressors and put in place appropriate action plans to manage these hazards, which in turn should ameliorate potentials for burnout and depression, as well as improve sleep quality.

Post-traumatic stress in firefighters was related to sleep quality through eight pathways. Definitions of PTSD indicate sleep disturbance is commonly present, however a new finding from the structural equation modelling is that burnout and depression modulates post-traumatic stress in terms of its impact on sleep quality. Critically, although exposure to trauma may be a part of the firefighter’s job, it also a part of employers’ duty of care to protect their employees. The range of scores from our survey indicated that some firefighters had a high number of symptoms and possible PTSD. On-the-job training for firefighters should include cognitive coping skills to manage any mental rumination of traumatic events experienced which typically occur during sleep times. There is a good business case for doing so: poor health indicators, presenteeism, absenteeism and leaving the profession – all of which are costly – emerge from poor management of PTSD (Kip et al. 2012; Wald and Taylor 2009).

The results showed that burnout has a direct role on sleep quality – replicating previous findings (Söderström et al. 2004) – and a modulating role. Its modulating effect for work-related stress on sleep quality, and post-traumatic stress on sleep quality, are new findings. Tackling all risks for burnout should be a priority for employers and line-managers, and particularly in firefighting where there are further consequences in terms of modulating post-traumatic stress and job stress, and in turn decreasing sleep quality. The relationship between emotional exhaustion and low sleep quality has previously been highlighted (Li et al. 2020), however in this study, we found that all burnout measures are important for understanding, and managing all aspects of burnout will ultimately improve sleep quality. Moreover, knowing that depersonalization had the strongest relationship of burnout and sleep quality in firefighters provides a focus for intervention. This aspect of burnout is characterised by detachment from co-workers, lack of motivation and a distorted sense of self. The impact of depersonalization on effective working has been discussed at length, most notably by Garden (1987). The salience of depersonalization in the manifestation of burnout has been highlighted by Wang et al. (2021) who ascribed a depersonalization score equal to or higher than 11 as related to severe burnout. The mean depersonalization score in this study was 11.34. This is indicative of a high prevalence of burnout in the sample which, if tackled, can provide a positive step for improving sleep quality.

A strength of our study is the large sample size, which provided sufficient statistical power for confidence in the analyses, and the use of valid and reliable measures in our survey instrument. We suggest our findings are robust enough to contribute to the development of interventions to ameliorate sleep problems in firefighters. Nevertheless, there were also some limitations around not collecting information regarding participants’ emotional health or family-related factors in relation to sleep schedules. Also, although localised recruitment is not unusual, and the firefighting job is similar across the globe, recruitment was limited to two provinces in Iran. Thirdly, the cross-sectional nature of the study is not as strong as a longitudinal design for determining causality between sleep disorders and related factors even using structural equation modelling. This should be explored further, although we suggest that a priority may be an intervention study that seeks to improve sleep quality in the firefighting profession.

## Conclusion

This study investigated the contribution of occupational stress to sleep quality in firefighters using structured equation modelling. The final model had an adequate fit to provide a trustworthy illustration that post-traumatic stress can affect sleep quality directly and indirectly through eight paths, involving burnout and depression as modulators, and work-related stress is related to sleep quality directly and indirectly through four paths in which burnout and depression are modulators. Burnout and depression are important variables for understanding sleep quality in firefighters, particularly as modulators of the impact of work-related stress and post-traumatic stress on sleep quality. Preventive measures for the high prevalence of poor sleep quality in firefighters should plan interventions that manage the risks associated with work-related stress and post-traumatic stress and the modulating parameters depression and burnout.

## Data Availability

The data generated during and analysed during the current study are not publicly available due to a denial of permissions from some of the fire stations who participated in this study. We must respect this right, as we are grateful for their co-operation within these parameters. We can make an anonymised database available from the corresponding author on reasonable request. Ethics approval, participant permissions, and all other relevant approvals were granted for this level of data sharing.
